# Analysis of morphine responses in mice reveals a QTL on Chromosome 7

**DOI:** 10.12688/f1000research.9484.2

**Published:** 2016-10-27

**Authors:** Wim E. Crusio, Esha Dhawan, Elissa J. Chesler, Anna Delprato

**Affiliations:** 1 Institut de Neurosciences Cognitives et Intégratives d'Aquitaine, UMR 5287, University of Bordeaux, Pessac cedex, France; 2 Institut de Neurosciences Cognitives et Intégratives d'Aquitaine, UMR 5287, CNRS, Pessac, France; 3 Lambert High School, Suwanee, USA; 4 BioScience Project, Wakefield, USA; 5 Jackson Laboratory, Bar Harbor, USA

**Keywords:** Opioids, QTL analysis, Locomotor activity, Reward pathway

## Abstract

In this study we identified a quantitative trait locus (QTL) on mouse Chromosome 7 associated with locomotor activity and rearing post morphine treatment. This QTL was revealed after correcting for the effects of another QTL peak on Chromosome 10 using composite interval mapping. The positional candidate genes are *Syt9* and *Ppfibp2*. Several other genes within the interval are linked to neural processes, locomotor activity, and the defensive response to harmful stimuli.

## Introduction

Responses to drugs of abuse vary among individuals and are genetically influenced ( Mistry *et al.*, 2014). Such drugs stimulate the brain reward pathway ( Gardner, 2011). Analysis of genetic variation in the behavioral and neurobiological processes can reveal molecular mechanisms that regulate or mediate the response to drugs, and neurobiological changes associated with chronic drug use. Many of the genes and signaling processes involved in the reward pathway in humans are conserved in mice ( Adinoff, 2004), and mouse genetic methods provide an efficient means of identifying these genes and loci.

Quantitative trait loci (QTL) analysis of recombinant inbred mouse strains (RIS) integrates phenotype and genotype data and is a widely used approach for studying the genetic basis of drug effects and addiction susceptibility ( Spence *et al.*, 2005). The BXD RIS, derived from C57BL/6J (B) and DBA/2J (D), are a well-established genetic reference population used in behavioral neuroscience studies for mapping complex traits associated with drug use ( Crabbe *et al.*, 1996; Dickson *et al.*, 2016; Gora-Maslak *et al.*, 1991; Peirce *et al.*, 2004; Plomin *et al.*, 1991).

In the present study we reanalyzed data from high throughput behavioral phenotyping of morphine-treated BXD RIS mice ( Philip *et al.*, 2010). Our purpose was to identify additional morphine response related genetic loci beyond those initially reported. We employed composite interval mapping for two behavioral phenotypes, rearing and locomotion in response to morphine, and identified a QTL on mouse Chromosome 7 that was masked by a major QTL on Chromosome 10.

## Methods

Experimental protocols have been described elsewhere ( Philip *et al.*, 2010). BXD data were generated in the laboratory of Dr. Charles D. Blaha at the University of Memphis and obtained from GeneNetwork.org.

### QTL mapping

QTL mapping was performed using GeneNetwork 1.0 with a composite interval mapping function using 2000 permutations. This is a forward regression approach in which a single locus with a major effect is included in a mapping model that scans for additional additive effects and interactions with the major locus. The technique will miss higher order interactions among loci that are not detectable as main effects, but is effective when a large, consistently observed major effect locus is present. Strain mean scores were Winsorized when statistical outliers were present ( Wilcox, 2005). We first determined which marker had the highest LRS value, using a marker regression analysis and then performed composite interval mapping, controlling for the Chromosome 10 SNP rs3721803, one of the 3 markers with the highest LRS scores.

### Bioinformatics

The MGI database ( http://www.informatics.jax.org/) was used to find information about SNPs and strain polymorphisms occurring within the Chromosome 7 interval (queried –June 20, 2016). This database now includes variants detected in the sequencing of 17 mouse genomes ( Keane *et al.*, 2011). The 3 SNPs located at the Chromosome 7 QTL peak (rs13479451, rs3724540, rs6386601) are at ~114.5 Mb in GeneNetwork and ~107.6 Mb in the MGI and NCBI databases ( http://www.ncbi.nlm.nih.gov/gene). In GeneNetwork, the relevant QTL interval on Chromosome 7 was 110–125 Mb whereas this region corresponds to 103–118 Mb in the MGI database. As finer mapping methods are developed, the exact interval location as reported here, may change. DAVID version 6.8 ( https://david.ncifcrf.gov/) was used to obtain functional annotations and pathway information for the genes within the interval and GeneWeaver 1.0 ( http://www.geneweaver.org/) to identify other drug-related phenotypes associated with the candidate genes.

## Results

### Composite interval mapping

**Figure 1.**
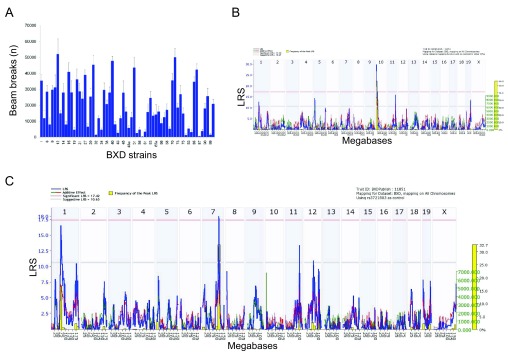
( **A**) Trait data for locomotion (number of beam breaks as a measure of distance traveled) for 64 BXD strains post morphine injection (Trait id 11851). ( **B**) Whole genome scan showing a robust QTL on Chromosome 10 (Trait id 11851, SNP rs372180). The x-axis represents chromosome number and megabase position and the y-axis represents the likelihood ratio statistic (LRS) of linkage. Blue lines indicate LRS across the genome. The pink and gray horizontal lines are approximate threshold values which are used to assess whether a peak is significant (P<0.05) or suggestive (P<0.63), respectively. Red and green lines represent the additive genetic contribution: red lines indicate negative values (C57BL/6J alleles increasing trait values) and green lines indicate positive values (DBA/2J alleles increasing trait values). Gray lines are shown when the parental strain is unknown. The yellow bars represent the relative frequency of peak LRS at a given location from 2000 bootstrap resamples ( **C**) Composite interval mapping of locomotion (Trait id 11851) after correcting for the peak on Chromosome 10 reveals a significant QTL on Chromosome 7.

As reported by Philip *et al.* (2010), whole genome scans produce robust QTLs mapping to Chromosome 10 at 0–30 Mb ( Figure 1A-B). The positional and functional best gene candidate is the *Oprm1* gene which encodes the opioid G-protein coupled receptor mu 1 ( Philip *et al.*, 2010) which has been previously detected ( Bergeson *et al.*, 2001; Doyle *et al.*, 2014).

We remapped these data for each time point after morphine injection (up to180 min) using composite interval mapping to identify QTLs potentially masked by this large QTL. This revealed a second significant QTL on Chromosome 7 for locomotion (LRS= 18.0, 114.7 Mb; Figure 1C) and, in an overlapping position, for rearing (LRS=16.5, 114.3 Mb). We also observed a time dependent decrease of the peak LRS values for both traits after the 60–75 minute time interval post-morphine injection. A suggestive QTL for the rearing trait data was already apparent on Chromosome 7 from the whole genome scan, but this was not the case for the locomotion trait data ( Philip *et al.*, 2010).

The area under the QTL peak contains 280 genes (MGI Chromosome 7: 103–118 Mb, GeneNetwork Chromosome 7: 110–125 Mb; database query-June 20, 2016, Supplementary worksheet 2). Of these genes, 162 are associated with olfactory receptors. Of the other genes with functional annotations, 11 are connected with neural processes ( *Adm:* neural tube development, *Appb1*, *Tub*, *Calca*, *Cckbr, Cnag4*: sensory perception and cognition, *Insc*, *Arntl*: neurogenesis, *Tpp1*: neuromuscular control, *Rras2*: regulation of neuron death and *Pde3b*: morphine addiction (KEGG pathway 05032).

There are 3 SNPs mapped at the location of the LRS peak and two positional candidates: Synaptogamin 9 ( *Syt9*: rs13479451, rs3724540) and PPFIA binding protein 2 ( *Ppfibp2*, rs6386601). Both of these genes differ between the parental strains (MGI database query – June 20, 2016). *Syt9* functions in vesicle traffic and Ca2+ triggered exocytosis ( Ncbi, 2016b) and *Ppfibp2* is involved in the regulation and development of neuronal synapses ( Ncbi, 2016a). These two candidates are also differentially expressed in the striatum of the brains of mice from strains with distinct opioid sensitivity ( Korostynski *et al.*, 2006; GeneWeaver: *Syt 9*, geneset #86830; *Ppfibp2*, geneset #86906).

## Conclusions

Genetic analysis of the BXD RIS resulted in the detection of an additional locus for morphine induced locomotor activity and rearing on Chromosome 7 which may be associated with activation of the reward system pathway in response to morphine treatment. Whether this effect is opioid-specific or also occurs with other classes of drugs is not yet clear. Alternatively, this QTL may also be related to general locomotor activity or nociception, a central nervous system response to potentially harmful stimuli. The best gene candidates within the QTL interval, besides the positional candidates *Syt9* and *Ppfibp2*, are *Calca* and *Cckbr* (sensory perception of pain) and *Pde3b* (morphine addiction).

## Data availability

The data referenced by this article are under copyright with the following copyright statement: Copyright: © 2016 Crusio WE et al.

All data are available in GeneNetwork ( www.genenetwork.org; see supplementary worksheet 1 for trait-ids and descriptions). Trait IDs: Locomotion: 11843, 11833, 11844, 11834, 11845, 11835, 11846, 11847, 11851, 11852, 11836, 11837, 11838, Rearing: 11884, 11885, 11886, 11887, 11888, 11878, 11854.

Data are also available in the Supplementary material.

## Supplementary material

GeneNetwork traits used in the QTL analysis.

.

List of genes within the Chromosome 7 interval.
