# Supplementary material for: Analysis of morphine responses in mice reveals a QTL on Chromosome 7
Source: F1000Res. 2016 Oct 27;5:2156. Originally published 2016 Sep 2. [Version 2] doi: 10.12688/f1000research.9484.2 (PMC5043443; doi:10.12688/f1000research.9484.2)
Supplement: Peer review history

# Peer review: Referee response for version 1

**DOI:** 10.5256/f1000research.10214.r16660

**Published:** 2016-09-28

**Authors:** Robert Gerlai

**Affiliations:** 1 Department of Psychology, University of Toronto, Mississauga, ON, Canada

I have only minor comments, listed below.

1. “In the present study we reanalyzed data from high throughput behavioral phenotyping of BXD RIS in the presence of morphine (Philip *et al.,* 2010).” Although it is clear what the authors are trying to say, strictly speaking this sentence implies that the authors had morphine present (on their desks? Or in their bloodstream??) when they were analyzing the data.  Please make the sentence less ambiguous.
2. “Strain mean scores were Winsorized when statistical outliers were present”. Please elaborate on this and/or cite references to support the decision to modify as opposed to exclude data.
3. “We remapped these data for each time point using composite interval mapping to identify QTLs potentially masked by this large QTL.” Please clarify what “time point” refers to here.
4. “protein coding genes” this is a redundant expression. Please use either “protein coding sequences” or “genes”.

 

 In the discussion, perhaps a few words could be mentioned about why in general reanalysis of prior phenotyping data may yield useful new pieces of information about QTLs..

I have read this submission. I believe that I have an appropriate level of expertise to confirm that it is of an acceptable scientific standard.

# Peer review: Referee response for version 1

**DOI:** 10.5256/f1000research.10214.r16166

**Published:** 2016-09-14

**Authors:** Clarissa Parker

**Affiliations:** 1 Department of Psychology and Program in Neuroscience, Middlebury College, Middlebury, VT, USA

The manuscript from Crusio *et al*. describes the use of GeneNetwork to identify QTLs associated with morphine response in a genetic reference mouse population.  The authors highlight many strengths afforded by both GeneNetwork and the BXD recombinant inbred mice.  Specifically, they used composite interval mapping to reanalyze publicly available phenotypic and genotypic data in the BXDs to identify a QTL on chromosome 7 that had been masked by a major QTL on chromosome 10. Next, they employed a bioinformatics approach to prioritize among candidate genes within the QTL interval.  This resulted in the identification of promising candidate genes that may be associated with drug-abuse phenotypes.

 One step that would strengthen the manuscript would be to use QTLminer in GeneNetwork to examine *cis*-regulation of the genes located within the QTL support interval (or even just the most promising candidate genes) and to see if any of those genes were expression QTLs in BXD mice.

 In addition, the authors raise an interesting question when they ask whether the chromosome 7 QTL is opioid-specific, or if it also occurs with other classes of drugs.  They could use GeneNetwork to search for genetic correlations between the expression levels of their top candidate genes and drug-related traits. 

 Minor Points:

 On the GeneNetwork website, Trait ID 11851 is defined as the number of beam breaks after morphine injection, but is described as “distance traveled” and “locomotion” in the Figure 1 legend of the manuscript.   

I have read this submission. I believe that I have an appropriate level of expertise to confirm that it is of an acceptable scientific standard.

# Response

**Published:** 2016-10-19

**Authors:** anna delprato

Reviewer comment: “One step that would strengthen the manuscript would be to use QTLminer in GeneNetwork to examine cis-regulation of the genes located within the QTL support interval (or even just the most promising candidate genes) and to see if any of those genes were expression QTLs in BXD mice.”

 Of the candidate genes there is a marginally significant cis-eQTL for Syt9 ( LRS  =18) but  this is with one probe and in the striatum dataset only.  There are no cis-eQTL for the other gene candidates.

 Reviewer comment: “In addition, the authors raise an interesting question when they ask whether the chromosome 7 QTL is opioid-specific, or if it also occurs with other classes of drugs.  They could use GeneNetwork to search for genetic correlations between the expression levels of their top candidate genes and drug-related traits.”

 Correlation analyses do not rule out or support association with other drugs. The few significant correlations that we found were based on less than 10 common strains.
